# Recruitment of the Host Plant Heat Shock Protein 70 by *Tomato Yellow Leaf Curl Virus* Coat Protein Is Required for Virus Infection

**DOI:** 10.1371/journal.pone.0070280

**Published:** 2013-07-23

**Authors:** Rena Gorovits, Adi Moshe, Murad Ghanim, Henryk Czosnek

**Affiliations:** 1 Institute of Plant Sciences and Genetics in Agriculture, Robert H. Smith Faculty of Agriculture, Food and Environment, Hebrew University of Jerusalem, Rehovot, Israel; 2 Department of Entomology, Volcani Center, Bet Dagan, Israel; Institute of Infectious Disease and Molecular Medicine, South Africa

## Abstract

A functional capsid protein (CP) is essential for host plant infection and insect transmission of *Tomato yellow leaf curl virus* (TYLCV) and other monopartite begomoviruses**.** We have previously shown that TYLCV CP specifically interacts with the heat shock protein 70 (HSP70) of the virus insect vector, *Bemisia tabaci.* Here we demonstrate that during the development of tomato plant infection with TYLCV, a significant amount of HSP70 shifts from a soluble form into insoluble aggregates. CP and HSP70 co-localize in these aggregates, first in the cytoplasm, then in the nucleus of cells associated with the vascular system. CP-HSP70 interaction was demonstrated by co-immunopreciptation in cytoplasmic - but not in nuclear extracts from leaf and stem. Inhibition of HSP70 expression by quercetin caused a decrease in the amount of nuclear CP aggregates and a re-localization of a GFP-CP fusion protein from the nucleus to the cytoplasm. HSP70 inactivation resulted in a decrease of TYLCV DNA levels, demonstrating the role of HSP70 in TYLCV multiplication *in planta*. The current study reveals for the first time the involvement of plant HSP70 in TYLCV CP intracellular movement. As described earlier, nuclear aggregates contained TYLCV DNA-CP complexes and infectious virions. Showing that HSP70 localizes in these large nuclear aggregates infers that these structures operate as nuclear virus factories.

## Introduction

Heat shock proteins 70 (HSP70s) are a conserved family of cellular chaperones that participate in a wide variety of cellular processes, including folding of newly synthesized polypeptides, refolding of misfolded or aggregated proteins, translocation of proteins, protein complex assembly or disassembly and protein degradation [1]. In addition to these diverse functions, HSP70s interact with a wide range of substrates and partners [1], [2], [3]. HSP70 chaperone activities rely on the energy of ATP to induce conformational changes in their substrates [1].

HSP70s are known to be associated with viral infections in animals and in plants [4]. Many plant viruses recruit host cell chaperones such as HSP70 to assist the synthesis, folding and localization of viral proteins, to regulate virus replication and to interfere with the antiviral host response ([5] and references therein). HSP70s together with their co-chaperones (mainly J-domain proteins) were shown to be associated with several processes in the viral infection cycle, including replication [6], [7], [8], assembly and disassembly of the viral capsid [9], [10], viral protein translocation, and viral protein association with membranes [11], [12]. During infection, HSP70s are involved not only in the regulation of the viral infection cycle, but also in the host stress response.

*Tomato yellow leaf curl viruses* (TYLCVs) consist of a group of begomoviruses (genus *Begomovirus*, family *Geminiviridae*) transmitted by the whitefly *Bemisia tabaci* [13]. TYLCVs infect the cultivated tomato (*Solanum lycopersicum*) and many other crop plants, ornamentals and weeds [14]. The TYLCV genome consists of one circular single-stranded genomic DNA of about 2.8 kb, which encodes six overlapping open reading frames (ORFs) [15], [16]. Two OFRs are located on the virus sense strand: V1 encodes the coat protein (CP), V2 - a movement protein with suppressor properties of RNA silencing. Four ORFs are located on the complementary viral strand: C1 encodes a replication-associated protein (Rep), C2 - a transcriptional activator protein, C3 - a replication enhancer protein, and C4 - a symptom and movement determinant. An intact CP is essential for cell-to-cell movement and systemic infection, nuclear import, particle formation, and transmission by the whitefly vector.

The interactions between geminivirus and plant host factors have been barely investigated, even though geminiviral proteins have a significant impact on cellular signal transduction pathways, DNA replication, cell cycle control and cell differentiation, plasmodesmata function and RNA silencing [17], [18]. For example, it has been shown that *Tomato golden mosaic virus* (TGMV) Rep protein interacts with the plant retinoblastoma homologue pRBR [19] to induce the transcription of the host enzymes necessary for TGMV DNA replication, since geminiviruses do not encode their own DNA polymerase [20]. Rep protein of *Tomato yellow leaf curl Sardinia virus* (TYLCSV) was shown to interact with the proliferating cell nuclear antigen (PCNA), which prevents DNA polymerase to dissociate from the viral template DNA strand, and recruits PCNA to the viral origin [21]. TYLCV V2 was shown to function as a viral suppressor of RNA silencing [22] through interaction with the host SGS3 [23] and papain-like cysteine protease CYP1 [24]. TYLCSV C2 interacts with CSN complex (COP9 signalosome; described in [25]) and regulates CSN5 activity on SCF E3 ligase and inhibits jasmonate signaling in *Arabidopsis thaliana* [26]. Since SCFs are key regulators of many cellular processes (SCF complexes are composed of Cullin1, S-phase kinase-associated protein, SKP1, the RING subunit RBX1 and F-box substrate binding protein), the ability of geminiviruses to selectively interfere with the activity of host proteins may represent a powerful strategy in the viral infection process.

Several lines of evidence support the possible involvement of plant host cellular chaperones, especially members of the HSP70 family, in the life cycle of geminiviruses. Silencing of the non-inducible form of HSP70, HSC70-1, resulted in impaired TYLCSV infection, supporting the hypothesis that production of this chaperone is required for geminivirus infection [27]. In higher plants, the best documented function of HSP70s in chloroplasts is the import of polypeptides into plastids [28], [29], which could be important for photosystem II protection and repair [30]. cpHSC70-1 was detected in the chloroplast stroma [31], in mitochondria and in nuclei in response to cold stress [32], [33], suggesting localization and possible functions in other cellular compartments. During synthesis, cpHSC70 may interact with the *Abutilon mosaic geminivirus* (AbMV) movement protein [34]. Silencing the expression of the plastid chaperone inhibited AbMV movement, but not viral DNA accumulation. Moreover, AbMV infection induced the subcellular re-distribution of cpHSC70-1 oligomers. A model was proposed describing how AbMV may utilize cpHSC70-1 for trafficking along plastids into a neighboring cell, or from plastids into the nucleus [35].

TYLCV is transmitted by its *B. tabaci* insect vector in a persistent circulative manner. It was proposed that insect proteins, especially at the midgut and salivary gland barriers, are involved in the recognition and translocation of the virus in the vector [36], [37]. The geminiviral CP was shown to be the only viral protein involved in insect-mediated transmission [38]. TYLCV CP interaction with a chaperonin GroEL homologue produced by *B. tabaci* endosymbiotic bacteria was required to protect virions from rapid proteolysis in the insect haemolymph [39]. A recent study profiled changes in the *B. tabaci* transcriptome in response to the acquisition and retention of TYLCV. Among the responsive genes identified, *hsp70* was shown to be induced upon virus infection. *In vitro* experiments confirmed the interaction of CP with HSP70 in *B. tabaci* protein extracts [40]. Feeding whiteflies with anti-HSP70 antibodies led to an increase in TYLCV transmission, suggesting that HSP70 restricts virus transmission, thereby protecting the insect from deleterious effects of the virus while translocating in the whitefly.

In this study, we investigated the association between TYLCV CP and the host HSP70 to gain insight on the possible role of this chaperone in the infection process in plants. The distribution, localization and interaction of TYLCV CP and the plant HSP70 were examined, and the results confirmed that the association between the two proteins in plant tissues is necessary for virus infection to proceed.

## Materials and Methods

### Sources of Virus, Insects and Plants

TYLCV [41] was maintained in tomato plants (cv. Daniella) by whitefly-mediated inoculation. Whiteflies (*B. tabaci* B biotype) were reared on cotton plants grown in insect-proof cages at 26°C, as described [42]. All plants were grown in a temperature-controlled greenhouse under standard rearing conditions. Tomato plants at their 3–5 true leaf stage were caged with viruliferous whiteflies (about 50 insects per plant at the onset of infection) for the duration of the experiments. Whiteflies were discarded before tissue sampling.

### Visualization *in situ* of TYLCV CP in Tomato Leaves and Stems

For histological analyses, cross sections of tomato leaves and stems (cut into 0.5 cm squares) were processed as described [43], [44]. Briefly, after fixation in 4% paraformaldehyde in MTSB (50 mM PIPES, 5 mM EGTA, 5 mM MgSO4, pH 7) leaf samples were embedded in wax (PEG 400 distearate and 1-hexadecanol – both from Sigma – mixed at a ratio of 9∶1). Fifteen µm-thick wax-embedded tissues were sectioned with a microtome (HM340E, Waldorf, Germany), rehydrated and blocked for 1 h at 25°C in 2% BSA/MTSB prior to incubation for 18 h at 4°C with anti-TYLCV-CP (raised as described in [45]) diluted 1∶100, and anti-HSP70 (Agrisera, Sweden) primary antibodies diluted 1∶200 in 2% BSA/MTSB. After washing with MTSB the samples were incubated for 1.5 h at 25°C with a Cy3- and Cy2-conjugated anti-rabbit secondary antibodies (Jackson Immunoresearch, USA) diluted 1∶200. The samples were inspected using a fluorescent microscope (Eclipse 80i, Nikon, Japan); CP and HSP70 were detected as red and green fluorescent signals, respectively. Plant nuclei were stained with 4′,6-diamidino-2-phenylindole (DAPI, Thermo Scientific DAPI, Pierce Protein Research Product), at 1 µg/ml for 20 min at 25°C, and detected as a blue fluorescent signal.

### Extraction and Fractionation of Proteins from Tomato Leaves and Stems

For analyses by SDS polyacrylamide gel electrophoresis (SDS-PAGE), protein samples from leaves and stems were prepared as follows. Leaves (pooled from three plants) were minced, frozen in liquid nitrogen and ground in a standard PAGE loading buffer supplemented with 2% SDS. Stems (3 cm-long pieces) were longitudinally cut and the stem content was extruded by exercising rolling pressure with a glass rod. Following centrifugation for 1 min at 1000 *g*, PAGE buffer was added to the pellet. Leaf and stem samples were boiled for 10 min and centrifuged for 40 min at 10,000 *g*; the supernatant was subjected to SDS PAGE. Parallel gels with the same samples were stained by PageBlue™ Protein solution (Fermentas).

Fractionation of proteins was performed by following previous reports [46], [47], [48], with modifications. Briefly, leaves (pooled from three plants) were collected and drill-homogenized in detergent-free buffer H (50 mM Tris-HCl pH 7.5, 80 mM KCl, 10 mM MgCl_2_, 0.2 mM EDTA, 1 mM dithiothreitol and Complete Protease Inhibitor Mixture - Roche, Mannheim, Germany). Stem pellet after spin down was re-suspended in buffer H and drill-homogenized. The leaf and stem homogenates were filtered through a cellulose membrane (N 334151, Schleicher and Schuell, Dassel, Germany) to separate debris and insoluble cell wall fraction from the filtrate. The latter was subjected to centrifugation at 3000 *g* for 20 min to obtain a pellet (P3) and a supernatant (S3). The insoluble cell wall fraction was re-suspended in PAGE sample buffer containing 4% SDS and boiled during 10 min. Proteins loosely bound to cell debris and insoluble proteins in the cell wall fraction were not separated further; the whole fraction was named Pellet (P).

Native total proteins were isolated as follows: leaves and stem spin down pellet (pooled from three plants) were drill-homogenized in buffer H supplemented with 0.5% Nonidet P40. Homogenates were incubated on ice for 45 min, vortexed and centrifuged at 1200 *g* for 10 min at 4°C. The supernatant containing native proteins was further analyzed. Cytosolic and nuclear protein fractions were prepared as described before [45].

### Sucrose Density Gradient Analyses

Extracts of native proteins (0.5 ml) were layered on 10 ml linear 10–50% sucrose gradients. After 20 h centrifugation at 104,000 *g* at 4°C (Beckman SW27 rotor), the gradients were fractionated into 10 aliquots as described previously [49]. For protein immunodetection, aliquots from each gradient were precipitated by ice-cold 10% TCA, washed with cold acetone and dried. Pellets were dissolved in SDS-PAGE buffer, boiled for 10 min and Western blot analyzed as described before [45]. Each gradient immunodetection was repeated at least five times.

### *In vitro* Immunodetection and Immunoprecipitation of Viral and Plant Proteins

Western blotting was performed as described [40], [45]. Incubation with first antibodies (anti-TYLCV, anti-HSP70 [Agrisera, Sweden] was followed by ECL detection (Amersham, UK). For immunoprecipitation, Protein A Sepharose beads (Pharmacia, Sweden) were incubated for 2 h at 4°C on a rotating shaker with an excess of either anti-CP or anti-HSP70 antibodies in binding buffer (50 mM tris-HCl pH 7.5, 150 mM NaCl, 2 mM EDTA, 0.5% NP-40). After unbound antibodies were removed, the protein extracts (400 mg) were added and incubated for 10 h at 4°C. Immunoprecipitated proteins were eluted by boiling in PAGE buffer. Each immunodetection was repeated at least three times for each set of plants (pooled tissues from three plants).

### DNA Extraction and qPCR Detection of TYLCV DNA

100 mg leaf tissues were ground with a drill homogenizer in 500 µl extraction buffer (100 mM tris HCl pH 8.0, 50 mM EDTA, 500 mM NaCl, 10 mM DTT, 2% polyvinyl-pyrrolidone). Samples were incubated with 10% SDS. After 15 min at 65°C, 160 µl of 5 M potassium acetate were added and samples were centrifuged for 10 min at 12,000 *g*. The supernatant was treated twice with an equal volume of phenol: chloroform (1∶1) followed by vortex and centrifugation at 12,000 *g* (4°C for 15 min), and by chloroform. DNA was recovered from the supernatant by ethanol precipitation and suspended in sterile distilled water. Samples were treated with RNAase A (25 µg/ml) for 20 min at 37°C, followed by inactivation at 65°C for 10 min.

TYLCV DNA was analyzed by qPCR in the presence of SYBR Green I (Takara, Japan) in a Corbett Research Rotor-Gene 6000 cycler. The reaction was as follows: 30 s at 94°C followed by 40 cycles consisting of 10 s at 94°C, 30 s at 59°C, and 20 s at 72°C. The primers used to amplify a 182 n fragment of TYLCV (GenBank X15656) were sense: 5′-TCTGTTCAAGGATTTCGTTG-3′ and complimentary sense: 5′-GCTGTCGAAGTTCAGCCTTC-3′ [50]. As an internal reference, a 183 n fragment of the tomato “Expressed” housekeeping gene (SGN-U346908) was amplified using the sense: 5′- CTAAGAACGCTGGACCTAATG-3′ and complimentary sense: 5′- TGGGTGTGCCTTTCTGAATG-3′ primers [51].

### Transient Expression of GFP-CP in *N. benthamiana*

*Agrobacterium tumefaciens* transformed with pBIN19-GFP-CP, provided by Dr. Gafni, Volcani Center, Israel [52], was used to infiltrate plant leaves. The bacteria were grown for 48 h at 28°C in LB medium containing 50 µg/ml kanamycin. The culture was centrifuged at 4,000 *g* for 8 min, and the pellet was re-suspended in water to a final optical density of 0.6 (OD 600). The suspension was used to infiltrate young leaves of 3-weeks-old plants by using a syringe. The GFP-fusion protein was visible under the fluorescent microscope 48 h after infiltration.

### Quercetin Treatment

Quercetin (Sigma, Israel) was dissolved in dimethyl sulfoxide (DMSO). Detached leaflets from infected tomato plants were placed in micro-tubes containing 400 µM quercetin (one leaflet per tube, the tip of the petiole soaking in the solution). The same volume of water in DMSO was used as control. The solutions were replaced daily for 4 days. For quercetin treatment of *Nicotiana benthamiana* leaves transiently expressing GFP-CP, 1 cm-diameter leaf discs were vacuum infiltrated with 800 µM quercetin dissolved in DMSO; DMSO containing the same volume of water served as control. The leaf discs were viewed under a fluorescent microscope (Eclipse 80i, Nikon, Japan), 2–4 h post infiltration.

## Results

### Concomitant Accumulation of CP and Decline of Cellular HSP70 in Tomato Leaves and Stem Upon TYLCV Infection

**Figure 1:**
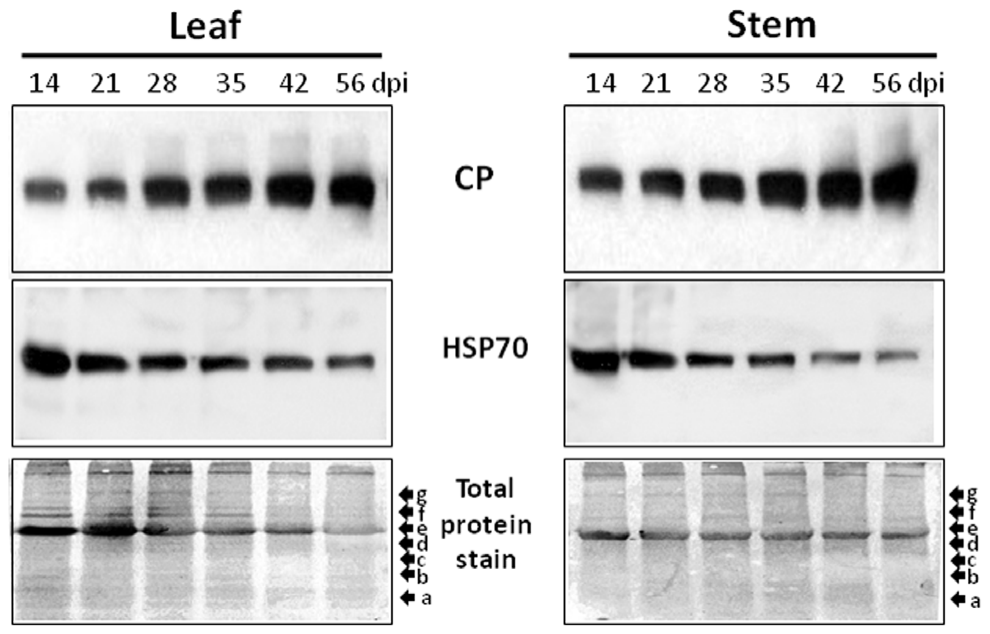
TYLCV CP and cellular HSP70 in total protein extracts of tomato leaf and stem during the progress of TYLCV infection. CP (upper panel) and HSP70 (middle panel) were western blot immuno-detected at 14, 21, 28, 35, 42 and 56 days after the onset of whitefly-mediated inoculation (dpi). Coomassie blue stain of total proteins (lower panel). Molecular weight markers a–g (in kDa): (a) 17, (b) 26, (c) 34, (d) 43, (e) 55, (f) 72, and (g) 95.

Tomato plants were caged with viruliferous whiteflies for up to 60 days. During this time, the accumulation of TYLCV CP was followed in total protein extracts from leaf and stem using an anti-CP antibody reacted. The 29 kDa protein (molecular mass in accordance with the translated V1/CP ORF) was first detected 14 days after the onset of infection (14 dpi) (Figure 1). In leaves and stem, the amount of CP increased with time, reaching a plateau at about 35 dpi and remaining steady for the next 30 days, until the plant started to decay. In contrast to the viral CP, the amounts of host HSP70 in leaf and stem decreased with the progress of virus infection - more pronounced in stem than in leaves (by comparison with total protein stain).

### Infection Brings Changes in the Solubility of HSP70

**Figure 2:**
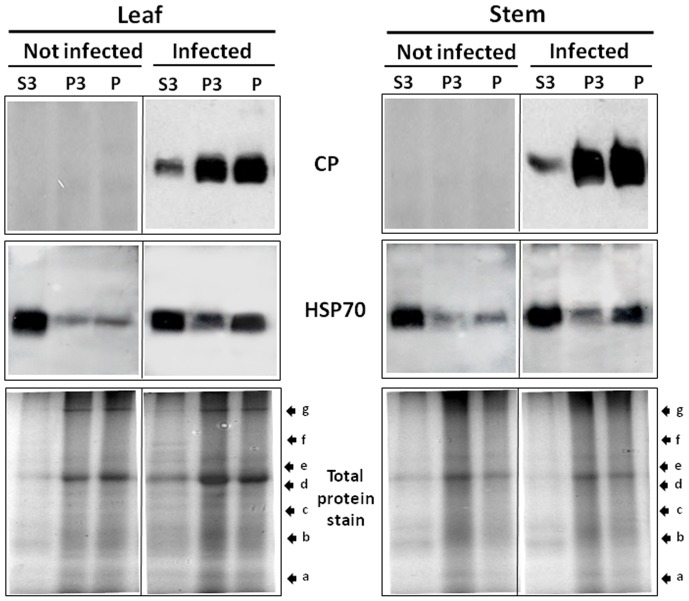
Viral CP and plant HSP70 in leaf and stem soluble and insoluble protein fractions. Western blot analysis of the leaf and stem homogenates from not infected and TYLCV-infected tomatoes were fractionated into insoluble debris and cell wall (P), 3000 *g* pellet (P3) and soluble protein (S3). Coomassie blue stain of total proteins (lower panel). Molecular weight markers a–g (in kDa): (a) 17, (b) 26, (c) 34, (d) 43, (e) 55, (f) 72, and (g) 95.

The association of viral CP and host HSP70 with soluble and insoluble fractions of tomato tissues was investigated. Leaves and stem at 28 dpi were fractionated under non-denaturing conditions into insoluble pellet (P3), soluble supernatant (S3) and cell walls and debris (P). The three fractions were analyzed by Western blot. In leaf and stem from non-infected plants, HSP70 was mainly found as a soluble protein (fraction S3), with lesser amounts in the insoluble fractions P and P3 (Figure 2). Upon virus infection, significant amounts of HSP70 became associated with insoluble aggregates (P3) and debris (P). CP was mainly detected in fractions P3 and P while only minor amounts were detected in fraction S3 (Figure 2). The presence of CP in P3 and P suggests that this protein is part of aggregates and inclusion bodies, either retained during filtration on the cellulose filter or sedimentation during centrifugation because of low solubility. The proportion of HSP70 in the insoluble fractions significantly increased (Figure 2) indicting that infection brought upon a change in the solubility of the chaperone.

### Upon Infection, HSP70 Co-sediments with TYLCV CP in Bottom Sucrose Gradient Fraction, Associated with Large Insoluble Aggregates

Further separation of soluble and insoluble leaf and stem proteins from non-infected and TYLCV-infected tomato plants was performed by ultracentrifugation on sucrose gradients (10–50%), in non-denaturing conditions. Leaf and stem filtrates were analyzed (fractions S3 and P3); cell wall fraction and cell debris (P) were discarded.

**Figure 3:**
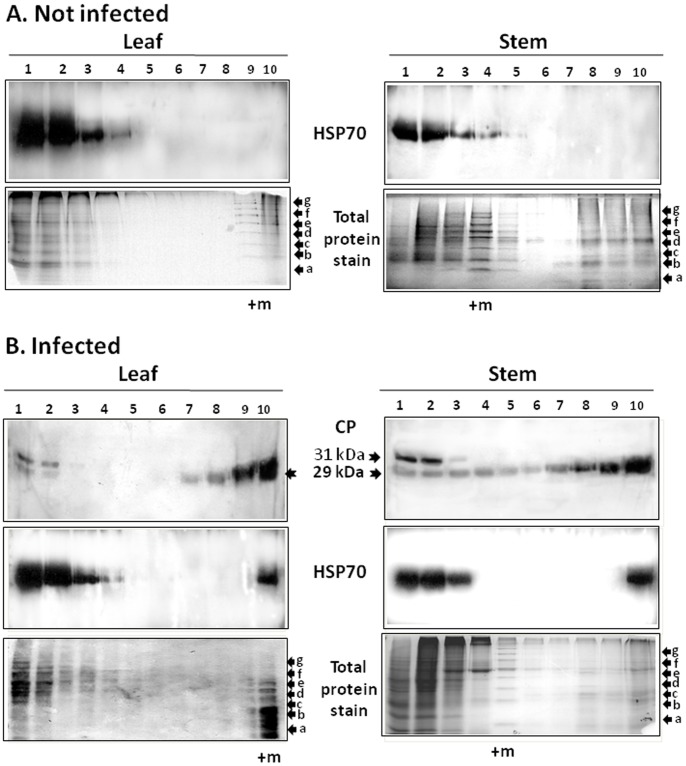
Distribution of viral CP and plant HSP70 following sedimentation on 10–50% sucrose gradients of native proteins extracted from tomato leaf and stem. Leaf and steam homogenates were prepared from tomato plants before (A) and at 28 days after the onset of TYLCV infection (B). Gradients were divided into 10 fractions, 1 (top) to 10 (bottom) and concentrated about 20 times by TCA precipitation (see Materials and Methods). Aliquots were subjected to SDS-PAGE. The gels were stained with Coomassie blue (total protein) and Western blotted using anti-CP and anti-HSP70 antibodies. Anti-CP recognized an additional minor 31 kDa polypeptide. Molecular weight markers (noted as m) a–g (in kDa): (a) 17, (b) 26, (c) 34, (d) 43, (e) 55, (f) 72, and (g) 95.

In gradients of leaves and stems of non-infected tomato plants, HSP70 was detected in gradient factions 1 to 4 (with small amounts in stem fraction 5), indicating that the chaperone was in a soluble form or in protein complexes (Figure 3A). HSP70 pattern changed upon infection. In leaf and stem, while most of HSP70 remained in factions 1 to 4, a significant portion of the chaperone was found in the bottom fraction (fraction 10), contained insoluble/aggregated proteins. In the same gradients, most of the CP from leaf and stem extracts was detected in fraction 10 (Figure 3B). A minor 31 kDa protein reacted with the anti-CP antibody in the fraction containing soluble proteins, the identity of which is unknown [45]. CP was also present in fractions containing small/midsized aggregates (7 to 9); in those fractions, CP was more abundant in the stem than in the leaf extracts. Moreover, in the stem but not in the leaf, CP was detected in fractions 3 to 6, suggesting that CP was present as monomers (top fractions), dimers, oligomers, up to high molecular weight aggregates (bottom fractions).

The obtained results showed that virus infection was accompanied by the presence of the cellular HSP70 and viral CP in the same gradient fraction (10), associated with large aggregates. However, HSP70 was not detected in fractions 7–9, contained small/midsized aggregates in both tomato tissues analyzed. The reason why HSP70 did not co-sediment with CP in those fractions is not known.

### TYLCV CP and Plant HSP70 Interact *in vitro*

**Figure 4:**
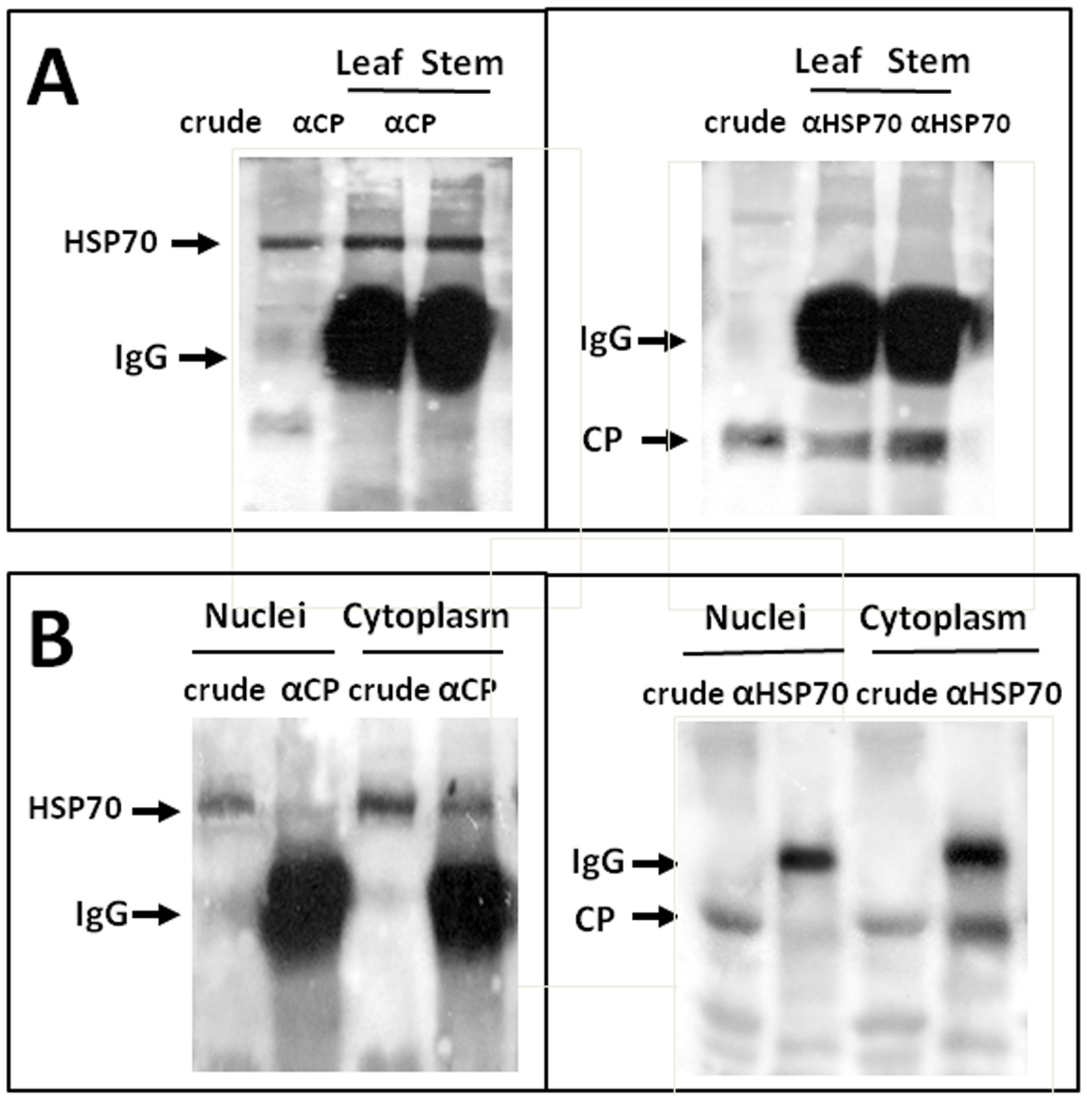
Co-immunoprecipitations of plant HSP70 by viral CP and *vice versa* in infected tomato tissues.

Sucrose gradient ultracentrifugation analysis showed that TYLCV CP and plant HSP70 co-sedimented in fractions 1–2 and 10 of both leaf and stem proteins and in additional fractions 3–4 from stem extracts (Figure 3B). The possible interaction between CP and HSP70 *in vitro* was tested by co-immunoprecipitation (Co-IP). CP-HSP70 complexes were pulled-down using anti-CP and anti-HSP70 antibodies and identified using anti-HSP70 and anti-CP, respectively (Figure 4A). The results showed that TYLCV CP formed complexes with cellular HSP70 in the tissues analyzed. Since the chaperone activity of HSP70 is ATP-dependent and controlled by successive cycles of ATP binding, hydrolysis and nucleotide exchange [53], the protein extracts were incubated with 8 mM ATP (30 min at room temperature) prior to immuno-precipitation. This treatment resulted in the disappearance of the CP-HSP70 precipitates (not shown) indicating that the formation of CP-HSP70 complexes was ATP-dependent. Co-IP performed as above indicated that CP-HSP70 complexes were detected in the cytoplasmic but not in the nuclear extracts (Figure 4B).

### TYLCV CP and Host HSP70 Co-localize in Aggregates of Increasing Size, First in the Cytoplasm then in the Nucleus of Infected Leaves and Stems

**Figure 5:**
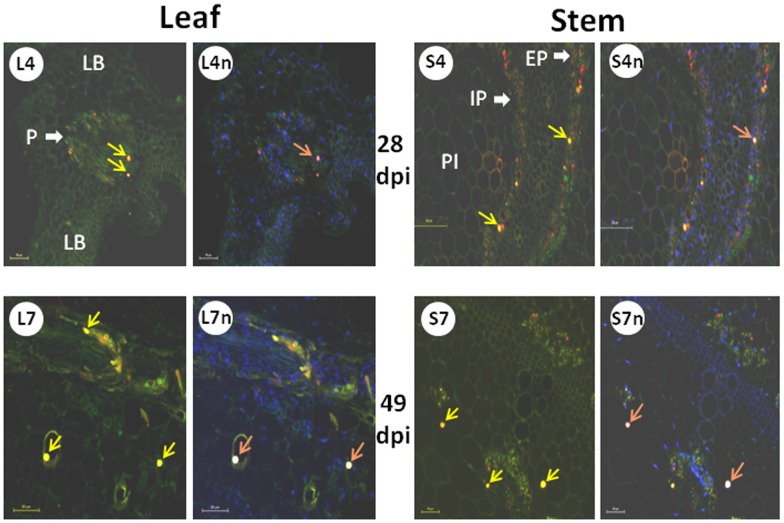
Co-localization of CP and HSP70 in cytoplasm and nucleus of infected tomato leaf and stem at 28 and 49 dpi. Leaf: cross-section through the midrib (P, phloem; LB, leaf blade). Stem: cross section between true leaves 2 and 3 (IP and EP, internal and external phloem; PI, pith). Fluorescent microscopy using primary anti-CP antisera and Cy3-labeled secondary antibody, primary anti-HSP70 antisera and Cy2-labeled secondary antibody; nuclei were DAPI stained. L4 and L4n: leaves 4 weeks after the onset of infection, without and with nuclei stain, respectively; L7 and L7n: same but 7 weeks after the onset of infection. S4 and S4n: stems 4 weeks after the onset of infection, without and with nuclei stain, respectively; S7 and S7n: same but 7 weeks after the onset of infection. Viral CP appears as red, cellular HSP70 as green, and nuclei as blue spots; CP in nuclei appears as violet, HSP70 in nuclei as light blue spots; CP co-localizing with HSP70 appears as orange to yellow spots (yellow arrows), CP co-localizing with HSP70 in nuclei - as pink to white spots (pink arrows). Bar: 50 µm, except for S4 and S4n, 100 µm.

CP was immuno-detected *in situ* in sections of leaves and stems collected from tomato plants at 28 dpi (when symptoms appeared) and at 49 dpi (clear disease symptoms) (Figure 5). CP was detected in phloem-associated tissues of leaves and stems. At 28 dpi, most of the CP fluorescence (red color) appeared as a mixture of small and larger dots. At 49 dpi, the CP signals were readily detectable and distributed in a punctate pattern, characteristic of aggregates/inclusion bodies. The intensity of the fluorescent CP signal increased in parallel with CP accumulation as shown by western blot analysis (Figure 1). These results demonstrated that the progress of virus infection was accompanied by the accumulation of increasing amounts of CP in infected leaves and stems, forming aggregates of increasing size.

HSP70 staining (green color) was also observed in phloem cells of leaves and stems (Figure 5). Moreover, co-localization of CP and HSP70 was already detected at 28 dpi in some of the small punctate fluorescent bodies (of yellow color). It was difficult to determine whether the fluorescence of soluble, not aggregated virus CP and plant protein HSP70 overlapped because of the low signal intensity. At 49 dpi, CP and HSP70 co-localized in aggregates of increasing sizes. Most of the large aggregates/inclusion bodies contained both proteins.

To distinguish between cytoplasmic and nuclear localization of CP-HSP70 aggregates, nuclei were stained with DAPI. Aggregates containing both CP and HSP70 were detected mostly in the cytoplasm at 28 dpi, but few in nuclei (pink to white spots, Figure 5). Three weeks later (49 dpi) large nuclear inclusions were conspicuous in leaf and stem. HSP70 did not appear in large aggregates/inclusion bodies in non-infected tomatoes (not shown). These results confirmed the co-localization of virus CP and host HSP70 in aggregates, which were re-localized into nuclei at the late stages of infection. Re-localization of aggregates from cytoplasm to nuclei correlated with an increase in the size of the aggregates, not only in leaf, but also in stem tissues.

### Inhibition of HSP70 Expression by Quercetin Changes TYLCV Accumulation and CP Localization in Plant Tissues

**Figure 6:**
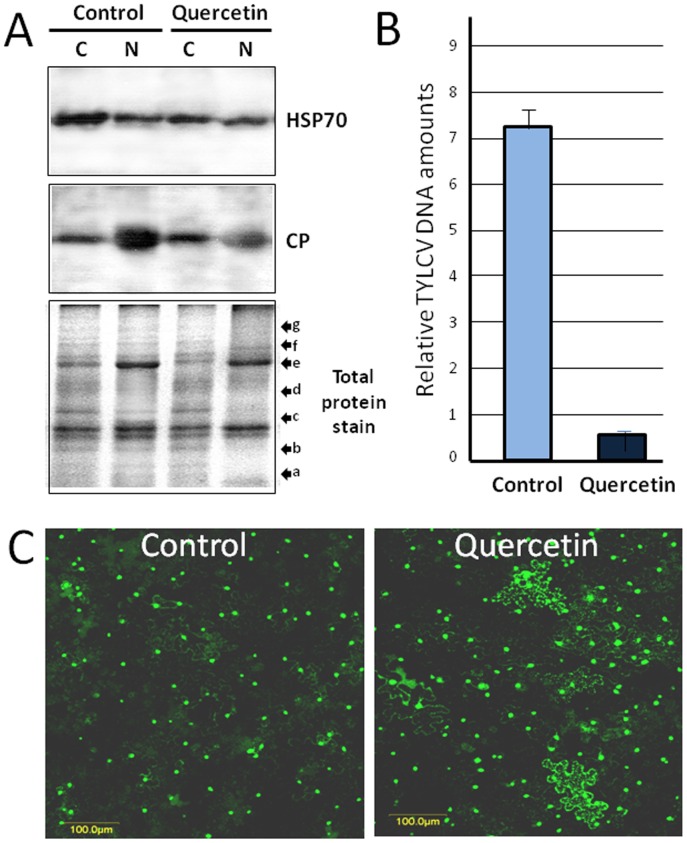
Changes affecting TYLCV and HSP70 in plants treated with quercetin. (A) Western blot analysis of leaf cytoplasmic and nuclear proteins from 28 dpi tomatoes before and after quercetin treatment (400 µM for four days). lower panel: Coomassie blue stain of total proteins; molecular weight markers a–g (in kDa): (a) 17, (b) 26, (c) 34, (d) 43, (e) 55, (f) 72, and (g) 95. (B) Tomato leaflets at 28 dpi were incubated for 4 days with 400 µM quercetin, TYLCV DNA amounts were estimated by qPCR analysis. (C) *N. benthamiana* epidermal cells transiently expressing GFP-CP following infiltration with quercetin (800 µM) or DMSO (control). Bar: 100 µm.

To determine whether cellular HSP70 plays a role in the localization of CP and consequently on TYLCV accumulation, we used quercetin, a bioflavonoid known to inhibit *HSP70* transcription in animal [54], [55] and plant cells [12]. Detached leaflets of infected tomatoes at 28 dpi were treated for four days with 400 µM quercetin dissolved in DMSO (DMSO alone was used as control). Under these experimental conditions, quercetin did not cause a visible damage to tomato leaves. Immunodetection of HSP70 in cytoplasmic and nuclear protein extracts showed that quercetin caused a minor decrease in the amount of HSP70 in the cytoplasm, but not in the nuclei (Figure 6A). Since the anti-HSP70 antibody used recognizes several members of the HSP70 family, we did not expect to obtain a complete inhibition of the expression of all HSP70s. Increase of the drug concentration to 1000 µM did not cause a complete loss of HSP70 (not shown). Immunodetection of viral CP in the same samples, showed a decrease in the amounts of nuclear CP in quercetin -treated tomatoes, while those of the cytoplasmic CP were similar to controls (shown for leaves in Figure 6A; comparable results were obtained with stems). Given that most of the viral CP was present in nuclear aggregates [45], we postulated that the decline in the amount of nuclear CP concerned its aggregated form.

The decline in the amount of nuclear CP in the treated tomato leaves was accompanied by a decrease of about 90% in the amount of viral DNA as determined by qPCR (Figure 6B). These results suggest the involvement of plant HSP70 in viral CP translocation from cytoplasm into nucleus. If translocation is impaired, the nucleus is depleted of TYLCV CP and the replication of genomic DNA is strongly inhibited.

The transport of CP from the cytoplasm to the nuclei was studied using discs of young leaves of *N. benthamiana* transiently expressing a GFP-CP fusion protein. The results showed that most GFP-CP accumulated in the nucleus (Figure 6C), as reported before [56]. In leaf discs, where HSP70 expression was inhibited by vacuum infiltration of 800 µM of quercetin, the fluorescence examined between 2 and 4 h post-treatment showed a subcellular re-distribution of GFP-CP from the nuclei to the cytoplasm (Figure 6C). Furthermore, cytoplasmic GFP-CP appeared as fluorescent punctate foci, but not as homogeneous staining. In control leaves (infiltrated with DMSO), no cytoplasmic fluorescent signals were detected. Thus, once HSP70 was inactivated, the GFP-CP fluorescence associated mainly with the nuclei changed its cellular localization to the cytoplasm.

## Discussion

For many plant viruses, a successful infection depends on the association between viral proteins and host chaperones (reviewed in [4]). HSP70s are central components of the cellular chaperone network, and are frequently recruited by viruses. HSP70 is also an essential component of the host defense response [57], including anti-viral response [58], [59]. Plant HSP70 has been shown to be induced in response to infection by viruses belonging to different genera (e.g. Carmovirus, Cucumovirus, Geminivirus Potyvirus, Potexvirus, Tobravirus) [60], [61], [62], [63]. Few studies have analyzed the expression of *hsp70* in various tissues after geminivirus inoculation by different means. Cotyledons excised from immature pea seeds were subjected to bombardment with microprojectiles coated with DNA extracts from sugar beet plants infected with *Beet curly top virus* (biolistic inoculation), a procedure much more aggressive than the natural leafhopper-mediated transmission. *In situ* hybridization of tissue sections at 3 dpi showed an increase in *hsp70* mRNA signals from a low level of constitutive expression in non-infected tissues. In this study, *hsp70* expression was not followed overtime [62]. In another study, the transcriptome (pyrosequencing reads) of pepper leaves infected with clones of *Pepper golden mosaic virus* delivered by biolistic inoculation, showed that a HSP70 chaperone was 2.6-fold up-regulated in symptomatic relative to mock-inoculated leaves [64]. We have analyzed various HSP70s in TYLCV-infected tomato plants using specific antibodies (HSP70/HSC70, cytoplasmic HSP70, chloroplastic HSP70 and ER HSP70/BiP). The results did not point to the accumulation of any HSP70s during the development of infection in leaves; on the contrary, the abundances of these key chaperones decreased during prolonged infection [65], [66] (Figure 1). On the other hand, the TYLCV-dependent decline of HSP70s was much less pronounced in tomatoes resistant than in tomatoes susceptible to the virus, indicating that resistant plants have a stronger chaperone homeostasis capacity to sustain virus stress than susceptible plants [67]. The results presented here indicate that HSP70-CP interaction facilitates CP translocation into the nucleus, thereby promoting replication and multiplication. This report analyzes some of the steps involved in this process.

During TYLCV infection, HSP70 is partially converted from a soluble into an insoluble form in tomato tissues (Figures 2, 3). Similar results were described for human *Respiratory syncytial virus* (RSV), which did not induce changes in HSP70 levels, but caused an increase in the abundance of membrane-associated HSP70 and the appearance of inclusions containing HSP70 [68]. In RSV-induced inclusions, viral polymerase complex was associated with cellular HSP70, which supported the possibility that inclusions are sites of RSV transcriptional activity. In the case of TYLCV, we have previously shown by *in situ* immuno-fluorescence microscopy that at early stages of infection, CP signals appeared as discrete punctate spots in the cytoplasm of phloem-associated cells [45]. With the progress of infection, the size of the cytoplasmic aggregates increased and large aggregates appeared in the nuclei. Nuclear aggregates containing the viral CP also included viral double stranded DNA replicative form, CP-DNA complexes and infectious particles, which could be transmitted to tomato test plants by whiteflies and induce the TYLCV disease. Here we showed that TYLCV infection induced the co-localization of HSP70 and CP first within large cytoplasmic aggregates, then with the progress of infection, in nuclear inclusions contained infectious particles (Figure 5). Co-IP demonstrated the capacity of HSP70 to form potential protein complexes with TYLCV CP in both leaf and stem (Figure 4A). The development of these complexes was ATP-dependent, suggesting that the chaperone activity is required in the interaction with viral CP. To demonstrate the contribution of HSP70 in intracellular CP translocation, Co-IP assays were performed with cytoplasmic and nuclear protein extracts. HSP70-CP interaction *in vitro* was shown for cytoplasmic, but not for nuclear proteins (Figure 4B), suggesting an important role for HSP70 in CP translocation from the cytoplasm to the nucleus. TYLCV CP has been shown to serve as a shuttle protein, mediating the import and export of DNA from cytoplasm into nucleus [69]. Hence we propose that HSP70 is one of the important components of the nuclear CP transportation complex. It has to be noted that such chaperones as HSP60 and BiP were not detected in large CP-containing nuclear aggregates (not shown).

Additional evidence for the HSP70 function in CP intracellular movement was demonstrated using quercetin, an inhibitor of HSP70 expression. It was previously shown that this drug induced protection against several RNA viruses belonging to different taxonomic families, including *Tomato bushy stunt virus*, *Cucumber necrosis virus*, *Turnip crinkle virus* and *Tobacco mosaic virus* [12]. We have followed the effect of quercetin on the localization of a GFP-CP fusion protein injected in tobacco leaves. In untreated leaves, GFP-CP localized in the nuclei [56] (Figure 6C). However, co-injection of the inhibitor of HSP70 expression with the fluorescent CP into tobacco leaves led to the re-localization of CP from the nuclei to the cytoplasm, suggesting that HSP70 is involved in the process of CP translocation to the nucleus. Application of quercetin to TYLCV-infected tomato caused a decrease in the amount of nuclear CP (Figure 6A) and, given that most of the viral CP is concentrated in large nuclear aggregates [45], to a decline in the development of nuclear inclusions. Since we proposed that the large nuclear aggregates function as virus factories containing infectious virions, a reduced aggregation should be accompanied by a decrease in the amounts of virus. Indeed, quercetin treatment of infected tomato leaflets caused a reduction of about 90% in the amount of TYLCV DNA (Figure 6B), indicating that HSP70 is required for TYLCV multiplication.

Several reports suggested a partial or complete dependence of virus survival on HSP70 [12], [27], [34]. However, our previous studies showing HSP70-TYLCV interactions inside the virus insect vector *B. tabaci*, revealed the opposite: TYLCV transmission was enhanced by whiteflies fed with anti-HSP70 antibodies [40]. Thus, the inhibition of HSP70-virus interaction/binding, which might help protect the insect from deleterious effects of the virus, allowed more TYLCV virions to pass the midgut barrier, to enter the haemolymph and to reach the salivary glands on their way to be transmitted to plants. These studies suggest that, in regard to the transport of TYLCV, HSP70 in plants and insect have opposite roles.

During plant infection, TYLCV CP is required for translocation to and out of plant nuclei, cell-to-cell movement, long-distance transport and viral replication [56], [69]. This implies a series of CP compatible interactions with host factors, such as chaperone HSP70. Results presented in the current study show that HSP70 is involved in CP-mediated nuclear transportation, followed by nuclear accumulation of viral DNA and the development of large nuclear viral inclusions that could serve as virus factories instrumental in ensuring an efficient TYLCV infection cycle. Since Co-IP was unable to demonstrate CP- HSP70 interaction in nuclear protein extracts, we propose that HSP70 and CP interact indirectly in nuclear CP inclusions *via* other viral components. HSP70 may be involved in the assortment of genome packaging and virion assembly, as suggested for non-inducible forms of HSP70 and HSC70, in mammalian orthoreovirus life cycle [70]. The HSC70 recruitment into orthoreovirus factory occurred via interaction with the virus matrix protein µNS and was independent of the chaperoning function of HSC70. Dominant negative mutants of HSC70 were found to localize in the µNS factories. Interestingly, HSC70 was readily detected in TYLCV induced nuclear inclusions (data not shown). A role for HSC70 in TYLCSV infection was recently proposed [27]. Silencing of HSC70 resulted in impaired TYLCSV infection, supporting the fact that chaperone production is required for a full geminivirus infection. One of the main features of virus factories in infected mammalian cells is the presence of chaperones (reviewed in [71], [72]). Thus, HSP70 localization in nuclear inclusions together with TYLCV CP, CP-DNA complexes and infectious virions supports the definition of these structures as nuclear virus factories [73], responsible for manufacturing TYLCV particles.

The role of HSP70 in long distance movement can be predicted because of the identification of HSP70-CP complexes in stem tissues of infected tomatoes (Figure 4A). Interestingly, it was shown that several HSC70 chaperones isolated from plasmodesmata (PD)-rich wall fractions could modify the PD size exclusion limit, promoting virus intercellular movement [74]. In tomato cell wall protein fractions, the abundance of HSC70 (not shown) and HSP70 (Figure 2) was very low, thus studying the HSP70 involvement in trafficking of TYLCV components through PD was not readily feasible. The ATP-dependence of HSP70-CP interaction raises the possibility that HSP70 could serve as a motor protein to facilitate the transport of virus-associated compounds through nuclear membranes and along the phloem, keeping the CP in a suitable folding state.

The current study shows how the cellular chaperone HSP70 can be used by the geminivirus TYLCV to achieve a successful infection. However, at the same time, HSP70 may target TYLCV components for degradation as part of the plant antiviral defenses. Discriminating between these two effects, which may occur in parallel, is the subject of further investigations.
